# Diabetes in Pregnancy: A Review of Service Provision and Practice at a Maternity Center

**DOI:** 10.3390/life16030410

**Published:** 2026-03-03

**Authors:** Hala Abdullahi, Suruchi Mohan, Chinnu George Samuel, Ibrahim Ibrahim

**Affiliations:** 1 Obstetrics and Gynecology, Sidra Medicine, Doha P.O. Box 26999, Qatar; habdullahi@sidra.org; 2 Grange University Hospital, Aneurin Bevan, University Health Board, Cwmbran NP44 8YN, UK; surchimohan@yahoo.com; 3 Clinical Trials Office, Sidra Medicine, Doha P.O. Box 26999, Qatar; cgeorgesamuel@sidra.org; 4 M/ACM Women’s Services, Doha P.O. Box 26999, Qatar

**Keywords:** gestational diabetes mellitus, hyperglycemia in pregnancy, clinical audit, care pathway adherence, postpartum OGTT screening, guideline implementation, Qatar, quality improvement

## Abstract

**Background**: The worldwide incidence of hyperglycemia in pregnancy is rising with the increasing prevalence of diabetes globally. In Qatar, Gestational Diabetes (GDM) is highly prevalent, at 31.6% of all pregnancies. Diabetes in pregnancy is associated with adverse maternal and fetal complications. This quality improvement practice project aimed to review the care pathway for managing diabetes in pregnancy with reference to the National Diabetes Guideline. **Method:** A retrospective audit was conducted through a chart review of electronic medical records for women who delivered at a tertiary maternity unit in Qatar, between 1 July 2022 and 31 December 2022. The audited criteria and standards were based on the National Guideline for Diabetes in Pregnancy in Qatar. **Results:** There were 737 deliveries during the study period. Overall, diabetes complicated 36.1% of births, and GDM was diagnosed in 34.6%. Of the patients with GDM, 197 (77.3%) were on diet control, 49 (19.2%) were on Metformin, and 9 (3.5%) were on Insulin. Pré-pregnancy weight was documented only in 140 (52.7%) women with diabetes. Of all the women with GDM, 52.9% were referred to see a dietician. Only 9 out of 164(5%) women with a BMI greater than 30 kg/m^2^ had an early Oral Glucose tolerance (OGTT). Orders for postpartum OGTT were found for 33.7% of the women with GDM. **Conclusions:** The audit identified some gaps in the care pathway for managing diabetes during pregnancy, particularly in documentation practices, nutritional therapy referrals, and postpartum diabetes screening. Addressing these gaps and overcoming the implementation challenges are crucial for enhancing care quality.

## 1. Introduction

The World Health Organization defines Gestational Diabetes (GDM) as hyperglycemia that is first detected in pregnancy and is neither type 1 nor type 2 diabetes [1]. With the prevalence of diabetes, even amongst young women, increasing worldwide and a rise in obesity and delayed childbearing, GDM has become the most common medical illness complicating pregnancy [2]. While recent global estimates of the prevalence of GDM are at 14% of all pregnancies, there is considerable regional variation, with the Middle East and North Africa (MENA) region having the highest prevalence, at 27.6% [3]. Qatar, a small, highly affluent Middle Eastern state where the present study is set, reported a GDM prevalence of 23.5% in 2018 [4], which rose to 31.6% when studied from 2019 to 2022 [5].

Hyperglycemia or diabetes in pregnancy can have significant repercussions for the short-term as well as the long-term health of mothers and babies. Pregnancy outcomes, including preterm labor, preeclampsia, macrosomia, shoulder dystocia, and cesarean delivery rates, are adversely affected by GDM [6]. Apart from increasing perinatal morbidity [7], GDM increases the risk of childhood obesity and future type 2 diabetes for babies born to affected mothers [8]. Mothers’ long-term health is also affected in the form of higher rates of diabetes and cardiovascular disease [9].

Studies have suggested that women with a history of GDM appear to have a nearly 10-fold higher risk of developing Type 2 Diabetes Mellitus (T2DM) than those with normal glucose in pregnancy [10]. Therefore, appropriate interventions in the early years after pregnancy are important to prevent the onset of T2DM. Champion et al. (2022) found that women diagnosed with GDM before 20 weeks are at higher risk for postpartum dysglycemia, underscoring the importance of follow-up [11].

Given the complex nature of diabetes and its broad ramifications for maternal and fetal health, meticulous management is critical, and a collaborative approach to care and prevention is recommended [12]. Further, healthcare stakeholders globally, including those from the USA [13], the UK [14], Canada [15], and others [16], have formulated several guidelines to aid optimum management.

As discussed, diabetes is a sizeable healthcare problem in Qatar, and the Ministry of Public Health has formulated guidance on the screening and management of diabetes in pregnancy [17]. Given the unabated rise in the prevalence of GDM in Qatar, the present study was undertaken as a quality improvement project to assess clinical care in compliance with this national guideline.

This project’s primary objectives were to review the existing care provision, identify potential adherence gaps to national recommendations, and propose improvements to optimize the quality of care provided. This article details the Audit’s findings, including demographic and prevalence characteristics, screening timing and strategies, treatment modalities employed, pregnancy outcomes, and postpartum care. The discussion of the results presented aims to provide insights into potential barriers to implementing the guidance and suggestions for improving the quality of diabetes care in the given context. It is hoped that this will inform healthcare stakeholders in the nation and the wider Middle Eastern region in healthcare planning to mitigate the significant regional burden of this disease

## 2. Materials and Methods

### 2.1. Study Design

This audit was conducted at a large, state-funded maternity and tertiary child health hospital in Doha, Qatar. Pregnant women with diabetes were identified through a retrospective review of electronic patient records for women who delivered at the hospital between 1 July 2022 and 31 December 2022. This study was conducted as a service evaluation/audit of clinical practice at a maternity center and did not constitute research involving human participants as defined by national regulations. Therefore, formal review and approval by an Institutional Review Board or Ethics Committee were not required.

### 2.2. Audit Standards

The Ministry of Public Health (MOPH), State of Qatar, published national guidelines on diabetes management in pregnancy in 2018, which served as the benchmark for this audit [17]. These guidelines outline diagnostic criteria and management pathways for diabetes in pregnancy. This audit specifically focused on the management of women diagnosed with GDM and those with pre-existing diabetes during pregnancy.

From this guidance, the following auditable criteria were adopted for this study:

1. Documentation of pre-pregnancy or early-pregnancy maternal weight
2. Women at high risk of GDM should have an early OGTT at 16–18 weeks of pregnancy.
3. On diagnosis of GDM, women should be referred to the dietitian
4. Women with pre-gestational diabetes should have an HbA1c check every trimester, receive 5 mg of Folic acid supplementation in the first trimester, receive aspirin in pregnancy, have a retinal assessment during current pregnancy, and be referred to an endocrinologist for review.
5. Patients with GDM should have an OGTT 6–12 weeks after delivery.

### 2.3. Data Collection

Electronic patient records were used to extract the demographic characteristics of the group, including age, parity, ethnicity, gestational age at booking and body mass index (BMI).

Relevant biochemistry data were collected, including early OGTT in women with a BMI greater than 30 kg/m^2^ and postpartum OGTT and documentation of referral to a dietitian and endocrinologist as applicable. Maternal and clinical outcomes, including preterm delivery, C-section, birth weight and gestational age at delivery, were extracted.

### 2.4. Outcomes

The project’s primary outcomes were to review the existing care pathway, identify gaps in adherence to national recommendations, and propose improvements to optimize the quality of care provided.

Secondary outcomes were the incidence of GDM, the rate of Cesarean section, and preterm birth among the study population.

### 2.5. Statistical Analysis

Data were analyzed using Microsoft^®^ Excel for Mac (Version 16.69, ©Microsoft 2022) and SPSS Statistics (Version 27.0, International Business Machine Corporation^®^, 2020). Categorical variables were expressed as a percentage for frequencies (with a 95% confidence interval), and continuous variables as mean ± standard deviation. Two-sample t-tests were used to compare continuous variables, and a chi-squared test was used to compare categorical data between the two groups.

Ethical approval was not required for this audit per organizational policy, as the data were anonymized and no identifiable patient information was accessed.

## 3. Results

**Figure 1:**
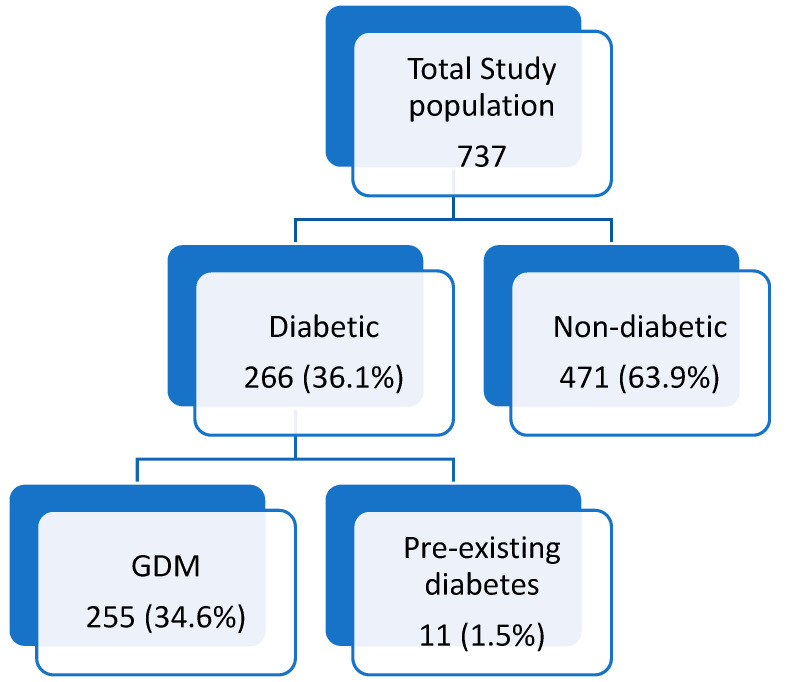
Distribution of diabetes diagnosis within the study population.

During the study period, 737 deliveries occurred, with diabetes diagnosed in 266 women (36.1%). Among these, 255 cases were classified as GDM, representing an incidence rate of 34.6%. Figure 1 illustrates the distribution of diabetes diagnoses within the study population. Among the GDM patients, 77.3% (197/255) were managed with diet control, 19.2% (49/255) required metformin, and 3.5% (9/255) were treated with Insulin. Table 1 displays the demographic characteristics of the study cohort. Women with diabetes (GDM or pre-existing) were significantly older than those without diabetes (mean age: 32.4 ± 5.2 vs. 30.5 ± 5.2 years; *p* < 0.001) and had a higher BMI (28.9 ± 5.3 vs. 26.8 ± 4.9; *p* < 0.001). Parity and ethnic origin were similar between the two groups, with no significant differences observed.

Pre-pregnancy weight was documented for 41% (302/737) of the entire cohort, with a higher documentation rate of 53% (138/266) among women with diabetes. As illustrated in Table 2, dietary service referrals were provided to 52.6% (140/266) of women with diabetes. Among those with GDM, 52.9% (135/255) were referred. Referral rates were similar between the GDM and pre-existing diabetes groups (52.9% vs. 45.5%). Of the 164 women with a BMI > 30 kg/m^2^, only 5% (9/164) underwent an early OGTT. Postpartum OGTT orders were placed for 33.7% (86/255) of all GDM patients. The likelihood of placing postpartum OGTT orders was higher in women treated with Insulin (66.7% [6/9]) or metformin (49.9% [24/49]) compared to those managed with diet alone (28.4% [56/197]. Compliance with the protocol for starting variable rate insulin infusion (VRII) during labor was 78% among women with pre-gestational diabetes and those with GDM treated with Insulin.

Table 3 summarizes some of the clinical outcomes of the study cohort. Cesarean section (CS) rates were significantly higher in women with diabetes compared to those without diabetes (45.5% [121/266] vs. 32.1% [151/471]; *p* < 0.001). Preterm delivery rates (<37 weeks) were nearly identical between the two groups (12.0% in women with diabetes vs. 12.1% in those without diabetes). The mean birth weight was 3.1 kg ± 1.4 in the non-diabetic group and 3.1 kg ± 0.5 in the diabetic group, with no significant difference between them.

Table 4 outlines the care of Type 1 and Type 2 diabetic women during pregnancy. Compliance with recommendations for folic acid supplementation (5 mg) in early pregnancy or preconception, HbA1c testing at each trimester, and retinal assessment during pregnancy was 100% in women with Type 1 diabetes. However, adherence to these recommendations was lower in women with Type 2 diabetes. The use of aspirin during pregnancy was reported in 50% of women with Type 1 diabetes and in 43% of those with Type 2 diabetes.

## 4. Discussion

This audit revealed gaps in managing patients with GDM in a high-diabetes-prevalence context. Specifically, pre-pregnancy weight was documented in only 52.7% of pregnant women with diabetes, limiting accurate BMI calculation and adherence to National and Institute of Medicine guidelines for gestational weight gain. The reasons for non-documentation were unclear. With the high prevalence of diabetes as discussed, the diabetes antenatal clinics are busy. Therefore, this non-documentation may possibly be due to staff oversight, with limited consultation times in busy clinics, or the patient’s inability to recall. Addressing this gap is critical for effective risk assessment and personalized care.

Early screening for diabetes in high-risk pregnant women, especially those with obesity, is crucial for timely GDM detection and management and therefore forms a part of guidance recommendations. The audit found that only 5% of women with a BMI over 30 kg/m^2^ received an early OGTT, highlighting a gap in screening adherence. This may be due to a lack of staff awareness of the guidance and therefore highlights a need for training/education efforts to align staff practice with recommended guidelines. Addressing this gap is essential for improving GDM detection and care in high-risk pregnant women in Qatar.

Effective GDM treatment reduces complications and promotes successful pregnancy outcomes [18]. Dietary management is key to appropriate gestational weight gain and should be part of care for pregnant women with diabetes. This is particularly important within a population with a high prevalence of diabetes and lifestyle-related metabolic disorders. The national guidelines recommend that all women diagnosed with GDM be referred to a dietitian. Our results showed that only 52.7% of women with GDM had a dietitian consultation, highlighting a gap in this essential aspect of care. Potential reasons for these low rates of referral shown may include a lack of awareness of guidance among staff, poor documentation, patient non-compliance, or gaps in patient understanding of the importance of dietary review. In addition, the low referral rates to the dietary service may stem from the fact that some women receive care from a mix of medical facilities, including private or public clinics, and may have chosen to have a dietitian review elsewhere, and this may not have been adequately captured or documented. The data also show that this referral is more likely in the first trimester of pregnancy. Emphasizing nutritional therapy in prenatal care is crucial for improving metabolic control and pregnancy outcomes. A systematic review demonstrated that nutritional therapy effectively reduces complications such as preeclampsia, excessive weight gain, and the need for Insulin or C-sections, as well as improves glycemic control [19]. Additionally, a retrospective cohort study found that medical nutritional therapy can reduce pregnancy complications, increase exclusive breastfeeding rates, and improve overall outcomes [20]. Therefore, these findings underscore the importance of staff and patient education.

With regard to the care of women with pre-pregnancy diabetes, the national guidance advises on the use of Folate 5 mg and aspirin in pregnancy, review by an endocrinologist, retinal examination, and an HbA1c check in each trimester. Upon reviewing the sample size for women with pre-existing diabetes (n = 11), we acknowledge that the small number of cases limits the statistical reliability of this criterion. Consequently, it would not be possible to draw meaningful inferences from the limited data for this subgroup. Further research with larger cohorts is required to better understand the management of pre-existing diabetes in pregnancy against the given guidance in the present setting.

Our study found a significant gap in postpartum diabetes screening, with only one-third of women receiving the recommended postnatal OGTT. A combination of factors may be at play here. In addition to staff oversight, where a postnatal GTT was not requested, it is also possible that some patients undertook this test at other private healthcare providers or with the state primary healthcare services, data for which would not be accessible to the study institution. An additional factor may be that the model of care at the study institution is one of paid services. Where patients did not internalize the importance of a rationale and the importance of a postnatal GTT, they would not be convinced to return for this test and/or to pay for it. Given the increased risk of type 2 diabetes and cardiovascular disease in women with a history of GDM, this is concerning. A meta-analysis revealed a 45% higher risk of cardiovascular disease in women with prior GDM [21]. These findings highlight the need for better postpartum screening and follow-up to reduce future health risks. The documenting of pre-pregnancy weight, early screening among high-risk women, dietitian referral, and postpartum diabetes testing are among the areas where this quality-improvement audit identifies practical gaps in adherence to national diabetes-in-pregnancy standards. By addressing these issues, automated reminder systems, staff training, and structured documentation tools may increase adherence to national standards and improve postpartum care continuity.

Postpartum follow-up is crucial for women with a history of GDM, but screening rates remain low. Contributing factors include a lack of awareness about long-term risks, prioritizing newborn care, time constraints, and inadequate guidance from healthcare providers. Research shows that improving GDM education and providing proactive care from diagnosis to postpartum follow-up can increase healthcare engagement among women with recent GDM [22]. Kilgour et al. (2019) identified confusion over guidelines and poor communication among healthcare providers as key reasons for low screening rates [23]. They suggested that midwives could play a role in improving communication and increasing postpartum GTT completion. Cochrane data review [24] showed that reminders significantly boost test uptake, raising rates from 14% with usual care to 57% with reminders sent to patients, providers, or both. Similarly, de Gennaro et al. (2020) found that recommendation letters to family physicians increased postpartum screening adherence from 41% to 47%, although challenges persist for younger or less educated women [25]. Overall consistency in maternal and newborn outcomes among pregnancies handled under standardized gestational diabetes treatment regimens was demonstrated by comparing the results with recent regional and international research.

These results might potentially guide the establishment of services in comparable high-prevalence environments in the Middle East and North Africa, where there is still variation in the application of standardized diabetes-in-pregnancy approaches.

### 4.1. Key Findings

The audit found several significant limitations in the present procedures that need to be addressed. Inconsistent pre-pregnancy weight documentation was discovered, which hindered precise risk assessment and early intervention planning. Despite the vital role that nutritional counseling plays in maximizing maternal and fetal outcomes, referral rates to dietary services were noticeably low. The inadequate uptake of early glucose tolerance testing (GTT) among individuals with elevated BMI suggests that possibilities for early detection and management of gestational diabetes risk were lost. The suggested postpartum oral glucose tolerance testing (OGTT) recommendations, which are crucial for identifying women at risk of developing type 2 diabetes after pregnancy, were also not well followed. All these results point to the necessity of more stringent adherence to screening standards throughout the continuum of treatment, better referral channels, and enhanced documentation practices.

### 4.2. Recommendations

Several specific changes are suggested to close the gaps found. To provide a thorough overview of each patient’s care and avoid missed interventions, documentation procedures should be standardized to guarantee consistent recording of important clinical metrics, such as pre-pregnancy weight, aspirin use, prior medical care, and any investigations or components of care undertaken at other institutions. The dietitian referral processes should be improved to promote prompt and effective nutritional care for individuals diagnosed with GDM. Postpartum screening protocols should be reinforced by developing dependable systems for reminders and follow-up to guarantee adherence to prescribed glucose tolerance tests. Additionally, to support complete, evidence-based diabetes management throughout the continuum of care and to reaffirm current guidelines, frequent monthly training sessions for providers are crucial.

## 5. Strengths

The audit showed several noteworthy advantages. It was backed by a sizable sample size that included 737 deliveries over a six-month period, improving the findings’ representativeness and dependability. The assessment was benchmarked against recognized standards of care since it was firmly based on adherence to the National Diabetes in Pregnancy Guidelines. The systematic identification of important elements in the management of diabetes during pregnancy was made possible by the application of clear evaluation criteria. Crucially, the audit produced results that could be put into effect, pointing out deficiencies in documentation and referral procedures along with useful suggestions for enhancement.

## 6. Limitations

When analyzing the results, it is important to consider the audit’s various constraints. Its retrospective design relied on the accuracy and completeness of existing records, which may have been insufficient in some circumstances. Because it was a single-center review, the findings might not be as applicable to other healthcare environments. There was insufficient contextual information since several areas, like the fundamental causes of low dietitian referral rates, were not thoroughly investigated. Furthermore, it was difficult to compare current practices with known benchmarks due to the small sample size of 11 pregnant women with diabetes. Diabetes long-term patterns or results pertaining to diabetes control during pregnancy might not be sufficiently captured by the very brief six-month assessment period. The retrospective single-center design, brief research duration, and small number of women with pre-existing diabetes are limitations of this study that might impact generalizability.

## 7. Conclusions

This quality improvement project has identified gaps in diabetes in pregnancy management in the setting of high diabetes prevalence. Implementing the recommended changes can significantly improve care for women with diabetes during pregnancy. Future audits and ongoing quality improvement initiatives are essential to ensure guideline adherence and improve patient outcomes.

## Figures and Tables

**Table 1:** Demographic composition of the study population.

Demographic Characteristic (N = 737)	No Diabetes 471	Diabetes (GDM, T1DM, T2DM) 266	*p*-Value	GDM 255	Pre-Existing DM 11	*p*-Value
Age (mean)	30.5 ± 5.2	32.4 ± 5.2	<0.001	32.3 ± 5.1	33.4 ± 6.2	0.475
Age (years) <18 18–35 >35	0 (0.0%) 383 (81.5%) 87 (18.5%)	0 (0.0%) 190 (71.4%) 76 (28.6%)	0.002	0 (0.0%) 184 (72.2%) 71 (27.8%)	00 (0.0%) 6 (54.5%) 5 (45.5%)	0.303
Parity Primipara Up to 3 4 and more	5 (1.1%) 385 (83.3%) 72 (15.6%)	2 (0.8%) 204 (77.3%) 58 (22.0%)	0.083	2 (0.8%) 197 (77.9%) 54 (21.3%)	0 (0.0%) 7 (63.6%) 4 (36.4%)	0.327
Ethnicity Nationals GCC Nationals Others	261 (55.4%) 18 (3.8%) 192 (40.8%)	161 (60.5%) 13 (4.9%) 92 (34.6%)	0.352	153 (60.0%) 13 (5.1%) 89 (34.9%)	8 (72.7%) 0 (0.0%) 3 (27.3%)	0.858
GA at booking	21.0 ± 11.6	16.6 ± 11.6	<0.001	16.8 ± 11.5	13.9 ± 12.8	0.418
BMI (kg/m^2^)	26.8 ± 4.9	28.9 ± 5.3	<0.001	28.8 ± 5.3	30.1 ± 6.0	0.451
BMI at booking <18.5 18.5–24.9 25–29.9 30 and above	8 (1.8%) 174 (38.5%) 171 (37.8%) 99 (21.9%)	0 (0.0%) 70 (26.6%) 88 (33.5%) 105 (39.9%)	<0.001	0 (0.0%) 68 (27.0%) 83 (32.9%) 101 (40.1%)	0 (0.0%) 2 (18.2%) 5 (45.5%) 4 (36.4%)	0.738

**Table 2:** Referral to dietetic services.

N = 737	No Diabetes (471)	Diabetes (GDM, T1DM, T2DM) (266)	*p*-Value	GDM (255)	Pre-Existing DM (11)	*p*-Value
Dietician referral	11 (2.3%)	140 (52.6%)	<0.001	135 (52.9%)	5 (45.5%)	0.626
Referral by GA at booking 1st trimester 2nd trimester 3rd trimester	10 (90.9%) 1 (9.1%) 0 (0.0%)	80 (57.14%) 44 (31.43%) 16 (11.43%)	<0.001 <0.001 <0.001	75 (55.56%) 44 (32.59%) 16 (11.85%)	5 (100%) 0 (0.0%) 0 (0.0%)	0.711 0.380 0.567

**Table 3:** Clinical outcomes.

N = 737	No Diabetes 471	Diabetes (GDM, T1DM, T2DM) 266	*p*-Value	GDM 255	Pre-Existing DM 11	*p*-Value
Preterm birth	57 (12.1%)	32 (12.0%)	0.977	29 (11.4%)	3 (27.3%)	0.133
C-Section	151 (32.1%)	121 (45.5%)	<0.001	113 (44.3%)	8 (72.7%)	0.064
Birth Weight (kg)	3.1 ± 1.4	3.1 ± 0.5	0.980	3.1 ± 0.5	3.1 ± 0.5	0.774
GA at delivery	38.5 ± 2.8	38.3 ± 2.2	0.222	38.3 ± 2.2	37.0 ± 1.0	0.055

**Table 4:** The care for Type 1 and Type 2 diabetic pregnant women.

Audit Standard	Type 1 (4 Patients)	Type 2 (7 Patients)
Folic acid 5 mg supplementation in early pregnancy/preconception	All 100%	5/7 (71%)
Aspirin during pregnancy	2/4 (50%)	3/7 (43%)
Referred to be seen by the Endocrinologist	100%	100%
HbA1c check in each trimester of pregnancy	100%	(86%)
Evidence of retinal assessment during pregnancy, whether it is done or advised	100%	2/7 (29%)

## Data Availability

The data that supports the findings of this study are available from the corresponding author on request.

## Abbreviations

The following abbreviations are used in this manuscript:

GDM	Gestational Diabetes Mellitus
T1DM	Type 1 Diabetes Mellitus
T2DM	Type 2 Diabetes Mellitus
OGTT	Oral Glucose Tolerance Test
BMI	Body Mass Index
HbA1c	Glycated Hemoglobin
VRII	Variable Rate Insulin Infusion
CS	Cesarean Section
GA	Gestational Age
MOPH	Ministry of Public Health
WHO	World Health Organization
IOM	Institute of Medicine
CVD	Cardiovascular Disease
**A summary of the Audit in context and key points:**	
**Category**	**Details**
1. What is already known about the subject?	1 Appropriate management of diabetes in pregnancy is critical for enhancing safe maternal and neonatal outcomes. 2 Adherence to national guidelines can improve outcomes but is often suboptimal.
2. What are the key questions?	1 Are current practices in line with the Qatar National Guideline for managing diabetes in pregnancy? 2 What gaps exist in the adherence to these guidelines? 3 How can the management of diabetes in pregnancy be improved at local setting?
3. What are the findings?	1 Significant gaps in guideline compliance, particularly in documentation and referral practices. 2 Low rates of dietician referrals and incomplete patient records.
4. How does this impact clinical practice for the foreseeable future?	1 Highlights the need for improved adherence to guidelines and better record-keeping. 2 Recommendations will enhance patient care and potentially improve outcomes for women with GDM.
